# Cetuximab and irinotecan as third-line therapy in advanced colorectal cancer patients: a single centre phase II trial

**DOI:** 10.1038/sj.bjc.6603018

**Published:** 2006-02-28

**Authors:** B Vincenzi, D Santini, C Rabitti, R Coppola, B Beomonte Zobel, L Trodella, G Tonini

**Affiliations:** 1 Medical Oncology, Campus Bio-Medico University, Via Emilio Longoni, 69, 00155 Rome, Italy; 2 Pathology, Campus Bio-Medico University, Via Emilio Longoni, 69, 00155 Rome, Italy; 3 General Surgery, Campus Bio-Medico University, Via Emilio Longoni, 69, 00155 Rome, Italy; 4 Radiology, Campus Bio-Medico University, Via Emilio Longoni, 69, 00155 Rome, Italy; 5 Radiotherapy, Campus Bio-Medico University, Via Emilio Longoni, 69, 00155 Rome, Italy

**Keywords:** cetuximab, irinotecan, advanced colorectal cancer, phase II trial

## Abstract

The epidermal growth factor receptor (EGFR), which participates in signalling pathways that are deregulated in cancer cells, is frequently mutated in colorectal-cancer cells. Cetuximab is a monoclonal antibody that specifically blocks the EGFR. We evaluated the efficacy of cetuximab in weekly combination with irinotecan in metastatic colorectal cancer patients refractory to previous treatments based on oxaliplatin or irinotecan. We included 55 heavily pretreated patients (colon/rectum: 34/11, M/F: 16/29, median age 63 years, range: 27–79) whose disease had progressed during or within an oxaliplatin-based first-line chemotherapy and a irinotecan-based second-line regimen. Patients were followed for tumour response and were also evaluated for the time to tumour progression, and safety of treatment. Cetuximab was given at an initial dose of 400 mg m^−2^, followed by weekly infusions of 250 mg m^−2^. Irinotecan was administered weekly at the dose of 90 mg m^−2^. All patients were assessable for treatment efficacy and safety response rate was 25.4% (95% CI: 21.7–39.6%); 38.2% (95 CI: 18.6–39.8%) of patients showed a disease stability as the best response. As a consequence, the overall tumour control rate was 63.6% (95% CI: 46.4–70.6%). The median time to progression was 4.7 months (95% CI: 2.5–7.1 months) and the median survival time was 9.8 months (95% CI: 3.9–10.1 months). The most common G3-4 noncutaneous side toxicities were: *diarrhoea* (16.4%), fatigue (12.7%) and stomatitis (7.3%). 89.1% of patients developed skin toxicity and 32.6% of cases was of grade 3–4. No allergic reactions were identified at any courses in any patients. Fever was documented in 27.3% of patients and was most commonly recorded after the first administration. Cetuximab has clinically significant activity even in heavily pretreated colorectal cancer patients progressed after both oxaliplatin and irinotecan-based chemotherapy regimens.

Colorectal cancer is the third most common cancer in the US, with approximately 145 000 new cases expected in 2005 (American Cancer Society, 2005). Estimated 5-year survival rates range from 90% for patients with stage I disease to <10% for patients with metastatic colorectal cancer (American Cancer Society, 2005).

Chemotherapy reliably enhances quality of life and prolongs both progression-free survival and overall survival (OS) for patients with metastic colorectal cancer (Grothey and Schmoll, 2001).

5-FU represented the mainstay treatment for patients with advanced CRC for a long period and no other drugs provided any real improvement in survival for CRC patients. However, several phase III trials investigating combination regimens with FU-LV plus irinotecan or oxaliplatin as first-line therapy have achieved an improvement of DFS and OS suggesting that combining these agents is advantageous (de Gramont *et al*, 2000; Douillard *et al*, 2000; Giacchetti *et al*, 2000; Goldberg *et al*, 2004).

Mainly owing to the introduction of irinotecan and oxaliplatin, in the past decade, the median duration of survival among patients with advanced colorectal cancer has increased from 12 months to about 18–21 months (Grothey *et al*, 2004). Moreover, the benefit of second-line chemotherapy has been clearly demonstrated by a randomised controlled trial in advanced colorectal cancer (Cunningham and Glimelius, 1999).

Chemotherapies, however, are limited by their lack of specificity and are often associated with frequent and potentially severe dose-limiting toxicities. Therefore, there is an urgent need for more effective, tailored and better-tolerated treatments that specifically target the processes pivotal to tumorigenesis and metastasis. Further advances in the understanding of molecular biology have led to the development of target-specific agents. The FDA recently approved two targeted agents: an antivascular endothelial growth factor (anti-VEGF) monoclonal antibody, bevacizumab and a human epidermal growth factor receptor (HER-1/EGFR) targeted monoclonal antibody, cetuximab as first and second-line metastatic colorectal cancer therapy, respectively (Venook, 2005).

Epidermal growth factor receptor (EGFR), a member of the ErbB family of receptors, is relevant in colorectal cancer because expression or upregulation of the *EGFR* gene occurs in 60–80% of cases. The EGFR signalling pathway regulates cell differentiation, proliferation, migration, angiogenesis, and apoptosis, all of which become deregulated in cancer cells (Salomon *et al*, 1995). Cetuximab is a chimeric IgG1 monoclonal antibody that binds to EGFR with high specificity and with a higher affinity than either epidermal growth factor thus blocking ligand-induced phosphorylation of EGFR. In addition, cetuximab enhances the effects of irinotecan and radiotherapy in experimental systems (Prewett *et al*, 2002).

A phase II study evaluated the activity and safety of weekly cetuximab plus irinotecan in patients with irinotecan-refractory CRC. The response rate was 17% in 121 patients, who had progressive disease on irinotecan (Saltz *et al*, 2001). A more recent phase II trial also assessed the safety and efficacy of single-agent cetuximab in patients with chemotherapy-refractory mCRC who express HER-1/EGFR (Cunningham *et al*, 2004). In this paper by Saltz and co-workers, 57 patients with EGFR-positive colorectal cancer that were refractory to both fluorouracil and irinotecan, were evaluated. The response rates were the following: 8.8% of the patients had a partial response to cetuximab monotherapy, and 36.8% had stable disease (Cunningham *et al*, 2004).

Cunningham and co-workers compared cetuximab alone *vs* cetuximab plus irinotecan in patients with irinotecan-refractory colorectal cancer in a phase III trial. The response rates were 10.8% for cetuximab alone and 22.9% for cetuximab plus irinotecan (14). Moreover, the 1-year survival rates in this group of heavily pretreated patients (29% in the combination-therapy group and 32% in the cetuximab monotherapy group) were encouraging (Saltz *et al*, 2004).

In the present phase II trial, we evaluated the efficacy and safety of the combination of cetuximab and irinotecan in EGFR-expressing colorectal cancer patients that progressed after oxaliplatin and irinotecan-based chemotherapies.

## MATERIALS AND METHODS

### Patients

We considered patients eligible if they were more than 18 years of age and had stage IV, histologically confirmed colorectal adenocarcinoma. In addition, immunohistochemical evidence of EGFr expression measured semiquantitatively (>0 on a scale of 0, 1+, 2+, or 3+) in a single reference laboratory (University Campus Bio-Medico, Rome). These measurements were performed and graded using a commercially available kit (EGFRpharmDx; Dako Corporation, Carpentino, CA, USA) according to the manufacturer's instructions. Patients were permitted to undergo the screening process for tumour EGFr expression before meeting other entry criteria and before study entry. For example, patients were allowed to undergo EGFr screening before study registration, while still receiving initial irinotecan therapy, before documentation of clinical progression. However, to be included in this study, patients were required before study entry to have demonstrated radiologic evidence of failure, as determined by the treating physician, on irinotecan or an irinotecan-containing regimen. Patients were not permitted to have received additional chemotherapy between the time of documented irinotecan failure and entry onto this clinical trial.

Other criteria for eligibility were: a ECOG performance-status score ⩽2, adequate haematologic function (haemoglobin ⩾9 g deciliter^−1^; neutrophil count ⩾1500 mm^−3^; platelet count ⩾100 000 mm^−3^), renal function (serum creatinine <1.5 times the upper limit of normal range), and liver function (total bilirubin <1.5 times the upper limit of normal range; aspartate aminotransferase and alanine aminotransferase <5 times the upper limit of normal values).

To be eligible, patients must also have previously received one oxaliplatin-based chemotherapy regimen (Capecitabine+Oxaliplatin or FOLFOX IV regimen, as first line) and one Irinotecan-based based-chemotherapy (FOLFIRI regimen, as second-line chemotherapy) for at least 2 months. All patients were included if progression of disease was documented during receipt of these regimens or within three months thereafter.

XELOX regimen was administered as following: Oxaliplatin 70 mg m^−2^ as continuous infusion for 12 h (0800–2000) on days 1, 8 plus chronomodulated capecitabine 1750 mg m^−2^ day^−1^ per os (0800 25% of total dose; 1800 25% of total dose; 2300 50% of total dose), on days 1–14 every 21 days (Santini *et al*, 2005).

FOLFOX IV consisted of LV (200 mg m^−2^ day^−1^) followed by a 5FU bolus (400 mg m^−2^ day^−1^) and 22-h infusion (600 mg m^−2^ day^−1^) for 2 consecutive days every 2 weeks with oxaliplatin 85 mg m^−2^ as a 2-h infusion on day 1 (de Gramont *et al*, 2000).

FOLFIRI consisted of CPT-11 180 mg m^−2^ as a 90-min infusion day 1; LV 400 mg m^−2^ as a 2-h infusion during CPT-11, immediately followed by 5-FU bolus 400 mg m^−2^ and 46-h continuous infusion of 2.4–3 g m^−2^ every 2 weeks (Andre *et al*, 1999).

Disease progression was documented by computed tomography (CT) or magnetic resonance imaging (MRI). At least one unidimensionally measurable lesion was required. EGFR expression in the primary tumour or in at least one metastatic lesion was performed, All the patients signed a consent form.

### Study design and treatment

This is a single centre phase II trial conducted from February 2004 – February 2005. Cetuximab was given at a loading dose of 400 mg m^−2^, followed by weekly infusions of 250 mg m^−2^. Irinotecan was administered weekly at the dose of 90 mg m^−2^.

A histamine-receptor antagonist and Atropine (0.25 mg) were given as premedication before every infusion. Moreover, dexamethasone was given at the dose of 20 mg before the induction course and at the dose of 8 mg in the further courses. A standard antiemetic drug was always given in the premedication and in the following days according to the physician's opinion. All the patients were to be treated until disease progression or unacceptable toxic effects occurred. In the case of disease progression, further anticancer treatments were allowed.

Tumour response was evaluated every 8 weeks with the use of consistent imaging techniques (CT or MRI). Assessment was performed by the investigators, who used the Response Evaluation Criteria in Solid Tumors (RECIST) (Therasse *et al*, 2000).

Toxic effects were assessed according to the National Cancer Institute Common Toxicity Criteria, version 2 (National Cancer Institute Common Toxicity Criteria, 1998).

Modifications of the dose of cetuximab were performed only in cases of toxic effects to the skin, and modifications in the dose of irinotecan were made in cases of haematologic or nonhaematologic toxic effects.

No grant support from Merck was given to anyone of the investigators and to the University Campus Bio-Medico.

### Statistical plan and analysis

A two-staged Simon accrual design was adopted for this phase II trial. The minimum target activity level was 20% and early discontinuation of the study was planned in the case of no response in the first 12 assessable patients. A planned sample size of 55 evaluable patients was chosen to better estimate efficacy. The primary end point was the rate of confirmed radiologic tumour response, as assessed by a local committee, in the intention-to-treat population. Secondary endpoints were the evaluation of time to disease progression (TTP), overall survival (OS), safety profile and median time to response. The median time of response duration was calculated from the date of response registration to the date of disease progression or death. All analyses were performed following an intention to treat analysis method. The time to progression was calculated as the period from the date of starting treatment to the first observation of disease progression or to death from any cause within 60 days after the start of treatment or the most recent tumour assessment. The overall survival time was calculated as the period from the date of starting treatment until death from any cause or until the date of the last follow-up, at which point data were censored. TTP and OS were both determined by Kaplan–Meier product-limit method (Kaplan and Meier, 1958).

Stratified permutation tests were carried out to explore the association between tumour response and rash and between tumour response and EGFR expression. Moreover, the difference in terms of TTP according to the presence and severity of acne-like rash was evaluated by the log-rank test (Peto *et al*, 1977).

The cut-off point for survival data was July 2005; for safety data, it was March 2005. SPSS software (version 11.05, SPSS, Chicago, IL, USA) was used for statistical analysis. A *P*-value of <0.05 was considered to indicate statistical significance.

## RESULTS

Between March 2004 and January 2005, 55 consecutive patients were enrolled in this single centre phase II trial; all of them had EGFR-positive tumors. The main characteristics of our patients population are summarised in Table 1. The median number of courses administered was 19 (range, 5–49 cycles). No protocol deviations were reported in any of our patients. All patients were evaluated for the declared study end points (tumour response, safety, TTP e OS).

### Efficacy analysis

**Figure 1:**
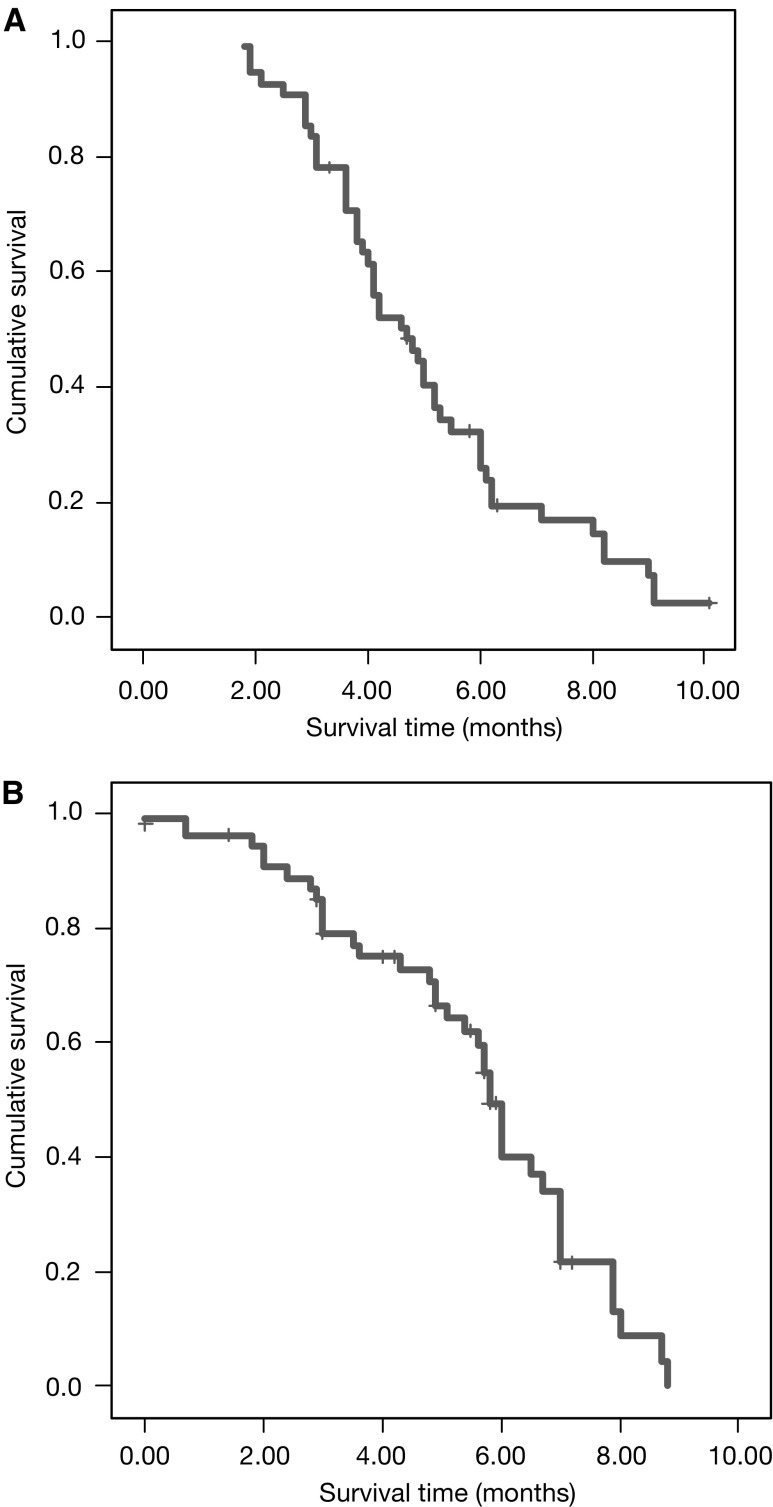
Kaplan–Meier survival plots for TTP (**A**) and OS (**B**) in advanced colorectal cancer treated with cetuximab plus irinotecan as third-line anticancer therapy.

For the intention-to-treat analysis, 55 patients were evaluated for efficacy. The best objective responses were achieved as follows: 0 (0%) complete responses (CR), 14 (25.4%; 95% CI: 21.7–39.6%) partial responses (PR), 21 (38.2%; 95% CI: 29.4–44.3%) stable disease (SD) and 19 (34.4%; 38.2%; 95% CI: 29.4–44.3%) disease progressions. Therefore, the overall response rate was 25.4% (95% CI: 21.7–39.6%), while the disease control rate (partial response+disease stabilization) was 63.6% (95% CI: 46.4–70.6%). The median duration of response was 4.9 months in the cohort of responding patients (95% CI: 2.1–8.2 months). The median TTP was 4.7 months (95% CI: 2.5–7.1 months), while the median OS time was 9.8 months (95% CI: 3.9–10.1 months). The survival curves are reported in Figure 1.

### Adverse events

All the 55 patients were included in the safety analysis. Skin reactions were observed in 49 patients (89.1%). In 70.9% (39 out of 55 patients) an acne-like rash, which is characteristic of cetuximab toxicity, was recorded. The other 10 patients (18.2%) with skin toxicity developed a mix of seborrheic dermatitis-like eruptions and maculopapular rash mostly on the face, trunk, and less frequently, on the limbs. In 16 patients (29.1%) skin toxicity was grade 3; all these patients were situated in the acne-like rash group. The median time of appearance of cetuximab-related skin toxicity was three weeks after the start of treatment (range: 1–8 weeks). The acneiform rash showed some degree of spontaneous partial improvement in the rash during the first 1 to 2 months of therapy and was subjectively noted in many of patients without modification of the cetuximab dose. Paronychial cracking was observed in 33 patients (60.0%). These lesions occurred either on the fingers or toes and usually evolved into chronic deep and painful lesions, in particular along the lateral surface of toes. Such lesions generally appeared later than the acne-like rash and were relatively persistent throughout the duration of the patient's treatment. After cetuximab interruption these lesions spontaneously regressed. No patients experienced allergic reactions leading to cessation of therapy.

Owing to persistent skin side effects cetuximab dose was reduced (25% dose reduction) in 10 patients (18.2%)

Leucopenia and neutropenia were the most common haematological toxicities with an incidence of 25.4 and 32.7%, respectively. However, neutropenia of grade 3–4 was recorded only in three patients (5.4%), but it did not cause any dose reductions or treatment discontinuation. No patients required administration of granulocyte colony stimulating factors to recover after neutropenic event. No neutropenic fever was recorded in our trial.

The most common nonhaematological toxicity were *diarrhoea* (grade 3–4 in 26.4% of patients), fatigue (grade 3–4 in 12.7% of patients), and stomatitis (grade 3–4 in 7.3% of patients). Owing to nonhaematological toxicities irinotecan dose was reduced (25% dose reduction) in 14 patients (25.5%). As a result of the persistence of *diarrhoea* in two patients irinotecan was discontinued and those patients completed the treatment only with cetuximab.

All the data about the safety profile are summarised in Table 2.

### Correlation of response and survival with rash

There was a correlation between the presence and severity of the acne-like rash and tumour response. In particular, patients with a grade *3 rah* showed an higher response rate (62.5%) vs those patients with a grade 0, 1 and 2 (10.25%); this difference is statistically significant (*P*=0.006). Moreover, comparing patients who developed a grade 3 acne-like rash with those who did not develop this toxicity or developed a grade 1 or 2, a statistical significant correlation was recorded between the acne-like rash and TTP (*P*=0.007). Moreover, a border line statistical significant difference in terms of overall OS between the two groups was identified (*P*=0.06).

**Figure 2:**
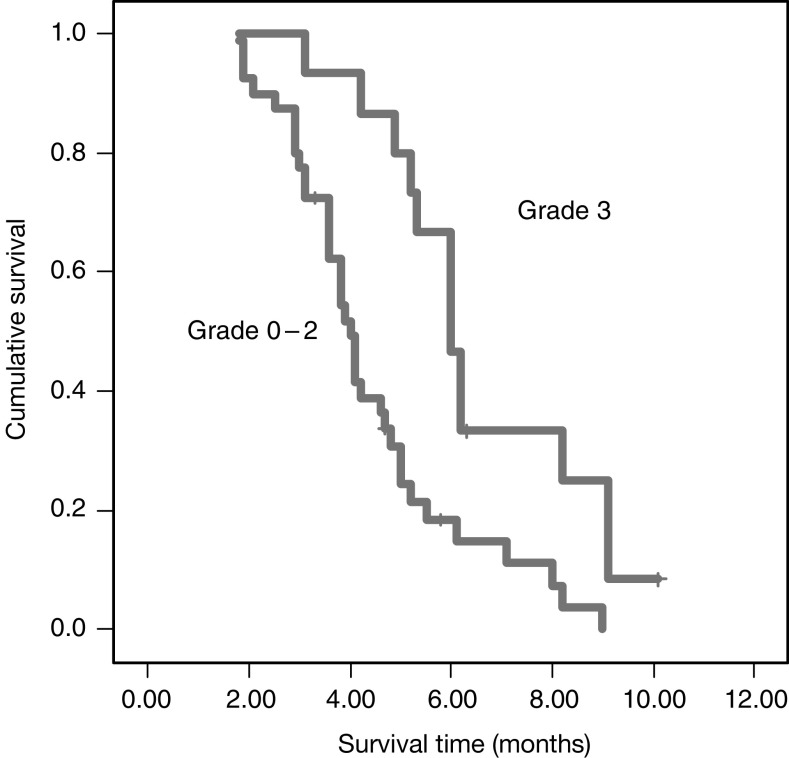
Kaplan–Meier survival plots for TTP in advanced colorectal cancer treated with cetuximab plus irinotecan as third-line anticancer therapy according to the presence and severity of acne-like rash.

These data are summarised in Table 3 and are shown in Figure 2.

### Correlation of response and survival with EGFR expression

We evaluated the correlation between the degree of EGFR expression and the response rate in all the 55 patients included in our analysis. Interestingly, we failed to identify a statistical significant correlation between the degree of EGFR staining and tumour responses. In particular, the distribution of tumour responses were: four partial response in the 16 patients with score 1+ (25%), four responses in the 20 with score 2+ (20%) and six responses in the 19 with score 3+ (31.6%) (*P*=0.708). Furthermore, no statistically significant differences in terms of both TTP and OS were identified according to EGFR staining (respectively, *P*=0.891 and 0.316).

## DISCUSSION

The epidermal growth factor receptor is a transmembrane glycoprotein that is involved in signalling pathways affecting cellular growth, differentiation, proliferation, and programmed cell death (Baselga, 2002). The receptor is present on the surface of normal epithelium and is overexpressed in certain tumors. Such overexpression has been associated with a poorer prognosis in colorectal cancer (Hemming *et al*, 1992; Mayer *et al*, 1993). Inhibition of this target can be achieved by antibodies directed against the extracellular domain of the receptor, by inhibitors of the dimerisation of the receptor or by small molecules that prevent phosphorylation of the receptor. Cetuximab is a monoclonal antibody against the extracellular binding domain of the receptor and recently became the first such inhibitor to be approved in the United States for the treatment of metastatic colorectal cancer (Andre *et al*, 1999).

Preclinical studies have shown not only that therapeutic synergy exists between cetuximab and chemotherapeutic agents, but also that such synergy can occur in tumour cells already resistant to irinotecan, a finding suggesting that the inhibitor may overcome cellular resistance to irinotecan (Baselga and Albanell, 2002).

We designed this trial to investigate the efficacy and safety of a combination of cetuximab with a weekly schedule of irinotecan. This is the first clinical experience of an association of cetuximab with a weekly schedule of irinotecan. In the paper by Cunningham *et al* (2004) patients received irinotecan at the same dose and schedule as that given during their most recent prestudy therapy. As a consequence of this trial design the included patients received a wide combination of irinotecan schedules.

Moreover, in this paper, the subset analysis revealed that cetuximab-based therapy was similarly effective in patients who had previously received oxaliplatin in addition to irinotecan before entering the study (Cunningham *et al*, 2004). On the basis of this result, we planned to include only patients that progressed also after an oxaliplatin-based regimen. As a result of all these reasons this trial is the first experience investigating the association of cetuximab plus weekly cetuximab as third-line therapy in advanced colorectal cancer patients.

Our results showed a response rate of 25.4% and a disease stabilization of 38.2%. Moreover, the median TTP was 4.7 months and the median OS was 9.8 months. These results are in their essence comparable with the previous data (Cunningham *et al*, 2004), confirming that a pretreatment with oxaliplatin does not represent a negative predictive factor for response to cetuximab plus irinotecan.

In the previous trials, the side effects of cetuximab are fairly mild, with an acne-like rash and drying and fissuring of the skin the most common; hypersensitivity infusion reactions are less frequent (occurring in 3% of patients, with death in fewer than 1 in 1000) (Cunningham *et al*, 2004). Although some degree of acneiform rash occurs in most patients, severe eruptions resulting in significant pain, pruritus, or infectious sequelae are rare. Of note, the development and severity of the rash have been correlated with an increased likelihood of an objective response.

In this trial, the association between the cutaneous toxicity and tumour response is statistically significant (*P*=0.006), confirming previously published data. However, for the first time, we investigated the impact of skin toxicity on TTP. This analysis led us to identify a statistically significant correlation of acne-like rash with TTP (*P*=0.007) and a border-line significant difference also in terms of OS.

The mechanism underlying the correlation between skin toxicity and tumour response is currently unclear, however, some research groups hypothesized that the rash is a surrogate indicator of an adequate degree of receptor saturation by cetuximab. If this is the case, then targeting doses to achieve a desired level of cutaneous toxicity may further increase the efficacy of this agent. While this is an appealing prospect from a potential efficacy point of view, it would suggest, if true, that there might be a narrow therapeutic window to work with for this agent (Perez-Soler and Saltz, 2005).

The trials reported to date have included only patients with immunohistochemical evidence of epidermal growth factor receptor expression. However, the degree of such expression appears to be unrelated to the likelihood of disease regression, raising questions as to whether receptor overexpression should be a prerequisite for cetuximab treatment and whether the drug interacts with additional molecular targets (Cunningham *et al*, 2004). More recently, Chung *et al*, (2005) reported that colorectal cancer patients with EGFR-negative tumors have the potential to respond to cetuximab-based therapies. Furthermore, in our experience the degree of EGFR expression did not represent a predictive factor for response and survival. As a consequence of all these observations, EGFR analysis by current immunohistochemical techniques does not seem to have predictive value (Dei Tos and Ellis, 2005).

In conclusion, this clinical trial provides the demonstration of a substantial clinical activity of the association of cetuximab plus weekly irinotecan as third-line treatment in oxaliplatin- and irinotecan-pretreated colorectal cancer patients. In particular, a previous treatment with an oxaliplatin based regimen does not seem to represent a negative predictive factor for response. Moreover, our data confirm that acne-like rash represents the only consistent predictive factor for response and identify, for the first time, a statistically significant impact of skin toxicity also in terms of TTP. Phase III trials should be designed to compare the association of cetuximab plus irinotecan *vs* best supportive care after oxaliplatin and irinotecan failure in advanced colorectal cancer patients.

## Figures and Tables

**Table 1:** Baseline characteristics of the patients

**Patient's characteristics**	**# of patients (%)**
Total number	55 (100%)
Male/female	21/34 (38.2%/61.8%)
	
*Age (years)*	
Median	63
Range	27–79
	
*Performance status*	
ECOG 0	26 (47.3%)
ECOG 1	18 (32.7%)
ECOG 2	11 (20.0%)
	
Primary tumour site	
Colon	38 (69.1%)
Rectum	17 (30.9%)
	
*No of metastatic sites*	
1	16 (29.1%)
2	26 (47.3%)
3+	13 (23.6%)
	
*Sites of metastases*	
Liver	33 (60.0%)
Lung	23 (41.8%)
Nodes	19 (34.5%)
Local	12 (21.8%)
Other	24 (43.6%)
	
*Prior adjuvant therapy*	
None	14 (25.4%)
FU/LV	32 (58.2%)
	
*First-line regimen*	
XELOX	38 (69.1%)
FOLFOX	17 (30.9%)
	
*Second-line regimen*	
FOLFIRI	55 (100%)
	
*EGFR expression*	
Score 1	16 (29.1%)
Score 2	20 (36.4%)
Score 3	19 (34.5%)

**Table 2:** Adverse events related to treatment

	**# of patients with toxicity (%)**	
**Side effects**	**All grades**	**Grade 3–4**
*Haematological*		
Anaemia	7 (12.7%)	2 (3.6%)
Leucopaenia	14 (25.4%)	0 (0%)
Neutropenia	18 (32.7%)	3 (5.4%)
Thrombocytopaenia	6 (10.9%)	0 (0%)
		
*Nonhaematological*		
Diarrhoea	28 (50.9%)	9 (16.4%)
Fatigue	26 (47.3%)	7 (12.7%)
Mucositis	21 (38.2%)	4 (7.3%)
Nausea/vomiting	18 (32.7%)	3 (5.4%)
Hypotension	9 (16.4%)	2 (3.6%)
Fever	15 (27.3%)	0 (0%)
Hypersensivity reaction	0 (0%)	0 (0%)
Acne-like rash	39 (70.9%)	16 (29.1%)

**Table 3:** Influence of acne-like skin rash on tumour response and survival

**Tumour response**	**# of patients (%)**	***P*-value**
Grade 0-1-2	6/39 (10.25%)	**0.006**
Grade 3	8/16 (62.5%)	
		
*TTP*	*Median TTP (95% C.I.) in months*	*P-value*
Grade 0-1-2	4.00 (3.6–4.4)	**0.007**
Grade 3	6.00 (5.3–6.7)	
		
*OS*	*Median OS (95% C.I.) in months*	*P-value*
Grade 0-1-2	9.1 (4.1–9.7)	0.06
Grade 3	10.3 (3.9–11.3)	

TTP: time to progression; OS: overall survival; 95% C.I.: 95% confidence interval.
